# Psoralen Suppresses Lipid Deposition by Alleviating Insulin Resistance and Promoting Autophagy in Oleate-Induced L02 Cells

**DOI:** 10.3390/cells11071067

**Published:** 2022-03-22

**Authors:** Yuhao Wang, Yonglun Wang, Fang Li, Jie Zou, Xiaoqian Li, Mengxia Xu, Daojiang Yu, Yijia Ma, Wei Huang, Xiaodong Sun, Yuanyuan Zhang

**Affiliations:** 1 West China School of Pharmacy, Sichuan University, Chengdu 610041, China; 2019224010004@stu.scu.edu.cn; 2 West China School of Basic Medical Sciences & Forensic Medicine, Sichuan University, Chengdu 610041, China; 2017151611022@stu.scu.edu.cn (Y.W.); 2019224010035@stu.scu.edu.cn (J.Z.); 2019224010037@stu.scu.edu.cn (X.L.); 2020224010013@stu.scu.edu.cn (M.X.); 2019151610004@stu.scu.edu.cn (Y.M.); 3 Department of Hepatopancreatobiliary Surgery, Sichuan Cancer Hospital and Institute, School of Medicine, University of Electronic Science and Technology of China, Chengdu 610041, China; 2120150970@mail.nankai.edu.cn; 4 Department of Plastic Surgery, The Second Affiliated Hospital of Chengdu Medical College, China National Nuclear Corporation 416 Hospital, Chengdu 610051, China; yudaojiang@cmc.edu.cn; 5 Department of Endocrinology, Affiliated Hospital of Southwest Medical University, Luzhou 646000, China; huangwei1212520@swmu.edu.cn

**Keywords:** psoralen, non-alcoholic fatty liver disease, autophagy, AMPK

## Abstract

Non-alcoholic fatty liver disease (NAFLD) held a high global prevalence in recent decades. Hepatic lipid deposition is the major characteristic of NAFLD. We aim to explore the mechanisms of psoralen on lipid deposition in NAFLD. The effects of psoralen on insulin resistance, lipid deposition, the expression and membrane translocation of glucose transporter type 4 (GLUT4), autophagy, and lipogenesis enzymes were determined on sodium oleate-induced L02 cells. Chloroquine and 3-MA were employed. The AMP-activated protein kinase alpha (AMPKα) was knocked down by siRNA. Psoralen alleviated insulin resistance in sodium oleate-induced L02 hepatocytes by upregulating the expression and membrane translocation of GLUT4. Psoralen inhibited lipid accumulation by decreasing the expression of key lipogenesis enzymes. Psoralen promotes autophagy and the autophagic flux to enhance lipolysis. Psoralen promoted the fusion of the autophagosome with the lysosome. Both chloroquine and 3-MA blocked the effects of psoralen on autophagy and lipid accumulation. The AMPKα deficiency attenuated the effects of psoralen on autophagy and lipid accumulation. Our study demonstrated that as an antioxidant, psoralen attenuates NAFLD by alleviating insulin resistance and promoting autophagy via AMPK, suggesting psoralen to be a promising candidate for NAFLD.

## 1. Introduction

The prevalence of non-alcoholic fatty liver disease (NAFLD) is on a steady upward trend worldwide in the last decades. The latest prevalence was estimated to be around 25.24% of the global population and 29.2% of the Chinese population [1,2]. NAFLD is mainly featured with insulin resistance and hepatic lipid accumulation, which encompasses a series of liver diseases, from simple hepatic steatosis to non-alcoholic steatohepatitis (NASH). It has been reported that about 59.10% of NAFLD patients evolve to NASH in 5 years and approximately 41% of the NASH patients progress to fibrosis and even cirrhosis [3]. Notably, among patients with NASH cirrhosis, the estimated annual incidence of hepatocellular carcinoma ranges from 0.5% to 2.6% [4,5].

Up to now, there are no effective medical interventions for NAFLD except for lifestyle modifications. Although vitamin E and pioglitazone have been demonstrated to be beneficial for NAFLD patients, the United States Food and Drug Administration hasn’t approved any specific drugs [6]. However, our previous studies have demonstrated that flavonoids such as naringenin and icaritin have obvious effects on ameliorating NAFLD [7,8]. As a kind of flavonoid, psoralen is the major antioxidant of Medik (syn. *Psoralea corylifolia* L) which is a traditional Chinese medicine termed “Buguzhi”. Clinically, psoralen along with ultraviolet A was used to treat hyperproliferative skin disorders such as psoriasis [9]. Psoralen was reported to ameliorate lipid accumulation in L02 cells [10,11]. However, the mechanism of psoralen ameliorating hepatic lipid accumulation remains unclear.

We aim to determine the effects and explore the mechanisms of psoralen on insulin resistance and lipid deposition in NAFLD. We established the NAFLD cell model by inducing L02 cells with sodium oleate. Then the impacts of psoralen on insulin resistance, lipid accumulation, lipogenesis (especially autophagy), and the activation of Adenosine 5′-monophosphate (AMP)-activated protein kinase (AMPK) pathway were determined. Our study demonstrated that psoralen ameliorates NAFLD by alleviating insulin resistance and promoting autophagy via AMPK. Since insulin resistance and hepatic lipid deposition contribute to several metabolic disorders such as type II diabetes and atherosclerosis, psoralen may serve as a candidate for metabolic diseases such as NAFLD.

## 2. Materials and Methods

### 2.1. Cell Culture and Treatments

The human hepatic L02 cell line was obtained from the American Type Culture Collection (ATCC), cultured with RPMI-1640 medium (Gibco, Waltham, MA, USA) supplemented with 10% fetal bovine serum (Biological Industries, Cromwell, CT, USA) and 1% penicillin-streptomycin (HyClone, Logan, UT, USA) at 37 °C and 5% CO_2_. When the confluence reached 40 to 50 percent, L02 cells were induced with sodium oleate at 100 μM for 24 h to induce lipid deposition. Then, L02 cells were stimulated with insulin (10 μg/mL) for 30 min and then treated with psoralen at 0.37, 1.1, and 3.3 μM for 24 h. Thereafter, glucose content was measured. To detect the effects of psoralen on autophagy, sodium oleate-induced L02 cells were treated with psoralen (3.3 μM) alone, or in combination with chloroquine (CQ, 10 μM), or 3-methyladenine (3-MA, 10 mM) for 24 h.

### 2.2. Glucose Content Assay

Glucose content was measured by the glucose content assay kit (Nanjing Jiancheng Bioengineering Institute, Nanjing, China). The culture medium was collected and mixed with the glucose assay solution. Then the mixture was transferred into a 96-well plate and determined by a SpectraMax 190 microplate reader (Molecular Devices, San Jose, CA, USA).

### 2.3. Western Blotting

L02 cells were digested by trypsin and lysed by sodium dodecyl sulfate-polyacrylamide gel electrophoresis (SDS-PAGE) loading buffer. Subsequently, samples were boiled at 98 °C for 10 min and proteins were separated on SDS-PAGE gel and transferred to a polyvinylidene fluoride (PVDF) membrane. Then, PVDF membranes were incubated with 5% skim milk (BBI Life Sciences Corporation, Shanghai, China) for 1 h. Thereafter, the PVDF membranes were incubated with primary antibodies at room temperature (RT) for 2 h or at 4 °C for 12 h. The following primary antibodies were employed: glucose transporter type 2 (GLUT2), glucose transporter type 4 (GLUT4), sterol regulatory element-binding protein 1 (SREBP1), fatty acid synthase (FASN), carnitine palmitoyltransferase 1a (CPT1a), LKB1, and phosphorylated LKB1 (p-LKB1) from Proteintech (Rosemont, IL, USA). AMPK, phosphorylated AMPK (p-AMPK), acetyl-CoA carboxylase (ACC), phosphorylated acetyl-CoA carboxylase (p-ACC), insulin receptor (INSR), phosphorylated insulin receptor (p-INSR), insulin receptor substrate 1 (IRS1), and phosphorylated insulin receptor substrate 1 (p-IRS1) were from Cell Signaling Technology (Danvers, MA, USA). Sequestosome 1 (p62), microtubule-associated protein 1A/1B-light chain 3 (LC3) from MBL (Nagoya, Japan), and glyceraldehyde 3-phosphate dehydrogenase (GAPDH) were from Santa Cruz (CA, USA). Thereafter, the PVDF membranes were incubated with a secondary antibody conjugated to horseradish peroxidase at RT for 1 h. Finally, blots were detected by enhanced chemiluminescence (Mei5bio, Beijing, China) and visualized by a chemiluminescence imager (Clinx, Shanghai, China).

### 2.4. Reverse Transcription PCR

We performed semi-quantitative reverse transcription PCR. Total RNA was extracted from L02 cell lysate by a TransZol Up kit (TransGen, Beijing, China) according to the manufacturer’s instructions. Then, cDNA was generated using super M-MuLV reverse transcriptase (Sangon, Shanghai, China), oligo(dT)12-18 primer (Sangon, Shanghai, China), and RNase inhibitor (BBI, Shanghai, China). The mixture was incubated at 50 °C for 45 min and reactions were terminated by incubation at 80 °C for 15 min. The cDNA was amplified using the following PCR conditions: 94 °C for 5 min, 30 cycles of 94 °C for 30 s, 60 °C for 30 s and 72 °C for 30 s, followed by 72 °C for 5 min, and 4 °C for 30 s. In addition, the PCR products were electrophoresed on 1% agarose gel. Finally, images were taken by ultraviolet exposure. Primers for SREBP1, FASN, CPT1a, β-actin, α-tubulin, and GAPDH are listed in Supplementary Table S1.

### 2.5. Immunofluorescence

L02 cells were fixed with 4% paraformaldehyde (PFA, Sangon, Shanghai, China) for 20 min. Then, cells were treated with 0.5% Triton X-100 for 15 min, blocked with 4% bovine serum albumin (BioFroxx, Einhausen, Germany) for 1 h, and incubated with anti-GLUT4 antibody (Sangon, Shanghai, China), anti-F-actin antibody (Thermo Fisher, Waltham, MA, USA), anti-LC3 antibody (MBL, Tokyo, Japan), or anti-lysosomal-associated membrane protein 1 (LAMP1) antibody (Santa Cruz, CA, USA) at RT for 2 h. Thereafter, cells were washed and incubated with the secondary antibody in darkness at RT for 1 h. Finally, nuclei were stained with Hoechst 33258 (Sigma, St. Louis, MO, USA) for 3 min after washing. Then, images were taken by confocal microscopy (Carl Zeiss, Jena, Germany) and the intensity of fluorescence was analyzed.

### 2.6. Nile Red Staining

L02 cells were stained with Nile red (5 μM) staining solution (Sangon, Shanghai, China) at 37 °C in darkness for 30 min. Then, cells were fixed with 4% PFA for 20 min after washing. Finally, nuclei were stained with Hoechst 33258 (Sigma, St. Louis, MO, USA) for 3 min. Pictures were taken by confocal microscopy (Carl Zeiss, Jena, Germany).

### 2.7. Plasmids and Transfection

GFP-double FYVE-containing protein 1 (DFCP1) plasmid was a kind gift from Professor Li Yu of Tsinghua University. The mRFP-EGFP-LC3 plasmid was obtained from Addgene (#21074). After L02 cells reached 60–70% confluence, cells were transfected with GFP-DFCP1 plasmid or mRFP-EGFP-LC3 plasmid using TurboFect transfection reagent (Thermo Fisher, Waltham, MA, USA) following the manufacturer’s instructions for 24 h. The transfection efficiency was observed via a fluorescence microscope (Carl Zeiss, Jena, Germany).

### 2.8. RNA Interference

After L02 cells reached 60–70% confluence, cells were transfected with control scrambled siRNA or AMPKα siRNA (Sangon, Shanghai, China) utilizing the TurboFect transfection reagent (Thermo Fisher, Waltham, MA, USA) for 72 h. The efficiency of knocking down the target protein, AMPKα, was evaluated by Western blotting.

### 2.9. Statistical Analysis

All experiments were conducted in triplicate. The gray value and fluorescence intensity were analyzed by ImageJ Version 1.53c. Data were expressed as the mean ± standard deviation (SD). The colocalization analysis was carried out by using the line-scan function of ImageJ. Representative cells were selected from each group and a 160 pixel-long line across the cell was drawn to scan the fluorescence dot on the cell membrane, cytoplasm, and nucleus. The fluorescence peak overlapping represented the colocalization. All experiment data were analyzed with a Student’s *t*-test by GraphPad Prism version 7.0. A *p*-value less than 0.05 was regarded as statistically significant.

## 3. Results

### 3.1. Psoralen Alleviates Insulin Resistance in Sodium Oleate-Induced L02 Cells by Increasing the Expression and Membrane Translocation of GLUT4

**Figure 1:**
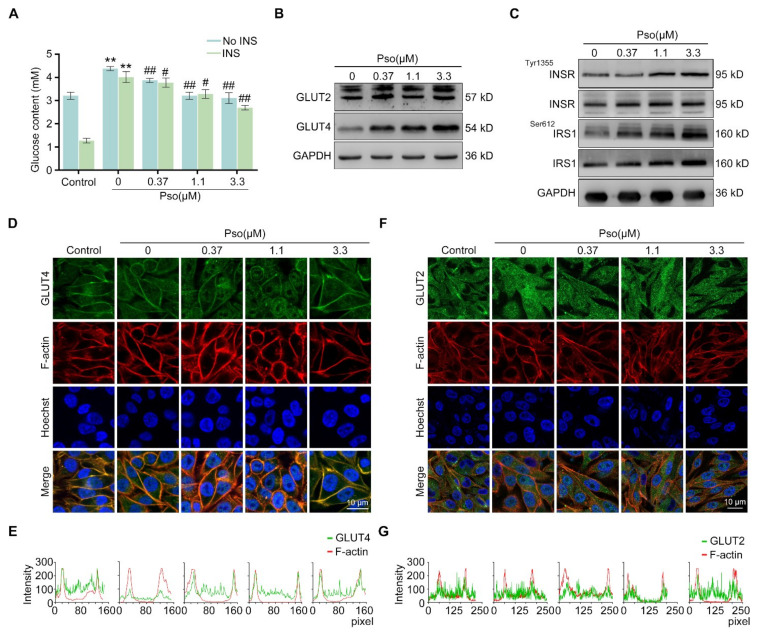
Psoralen alleviates insulin resistance in sodium oleate-induced L02 cells by enhancing the expression of GLUT4 and promoting the GLUT4 membrane translocation. L02 cells except the control group were induced with sodium oleate (100 μM) for 24 h to induce lipid deposition. Then, all cells were stimulated by insulin (10 μg/mL) for 30 min and then incubated with psoralen at 0, 0.37, 1.1, and 3.3 μM for 24 h. (**A**) The glucose content was measured by the glucose content assay kit. (**B**) The expression of GLUT4 and GLUT2 was detected by Western blotting. (**C**) The expression of INSR, p-INSR, IRS1, and p-IRS1 were measured by Western blotting. (**D**) The expression of GLUT4 and F-actin was detected by immunofluorescence and images captured by confocal microscopy (scale bar = 10 μm). (**E**) Colocalization efficiency of GLUT4 and F-actin in multiple cells (*n* ≥ 3 cells) within the field of view was quantified by line scan analysis (160 pixels with two ends on the membrane) by observing the overlap of fluorescence intensity peaks along the spanning contour. (**F**) The expression of GLUT2 and F-actin was detected by immunofluorescence and images captured by confocal microscopy (scale bar = 10 μm). (**G**) Colocalization efficiency of GLUT2 and F-actin in multiple cells (*n* ≥ 3 cells) within the field of view was quantified by line scan analysis (250 pixels with two ends on the membrane) by observing the overlap of fluorescence intensity peaks along spanning profiles. All values were expressed as the mean ± SD from three independent experiments. # *p* < 0.05, ## *p* < 0.01, vs. the control group; ** *p* < 0.01 vs. sodium oleate-induced group. Abbreviations: INS, insulin; Pso, psoralen; INSR, insulin receptor; IRS1, insulin receptor substrate 1.

The NAFLD cell model was established by inducing L02 cells with sodium oleate (100 μM) for 24 h. As shown in Figure 1A, control group cells exhibited decreased glucose content upon insulin stimulation. Compared with the control group cells, the 0 μM group cells exhibited much higher glucose content upon insulin stimulation, indicating that insulin resistance was established in sodium oleate-induced cells. Notably, psoralen significantly reduced the glucose content of sodium oleate-induced L02 cells in a dose-dependent manner. Since GLUT4 plays important role in glucose utilization, decreased location of GLUT4 on the cell membrane was reported to induce insulin resistance [12]. While GLUT4 is found primarily in adipose tissues and striated muscle, GLUT2 is a major isoform in the liver and insulin-independent. We measured the expression of GLUT4 and GLUT2 by Western blotting. As shown in Figure 1B, psoralen significantly upregulated the expression of GLUT4 at 0.37, 1.1, and 3.3 μM in a dose-dependent manner, while the expression of GLUT2 was not significantly changed by psoralen. Therefore, the expressions of several proteins including insulin receptor (INSR), p-INSR, insulin receptor substrate 1 (IRS1), and p-IRS1 in the insulin signaling cascade were detected by Western blotting. Psoralen enhanced the ratio of p-INSR/INSR and p-ISR1/ISR1 in a dose-dependent manner (Figure 1C). Colocalization analysis showed that sodium oleate significantly decreased the colocalization of GLUT4 and F-actin as detected by immunofluorescence (Figure 1D,E). Psoralen significantly enhanced the colocalization of GLUT4 and F-actin, especially at 3.3 μM, compared with sodium oleate-induced L02 cells. In contrast, the expression of GLUT2 and colocalization with F-actin were not changed by either sodium oleate or psoralen compared with control cells (Figure 1F,G). These results indicated that psoralen ameliorates sodium oleate-induced insulin resistance by increasing GLUT4 expression and membrane translocation.

### 3.2. Psoralen Alleviates Lipid Accumulation by Reducing Lipogenesis

**Figure 2:**
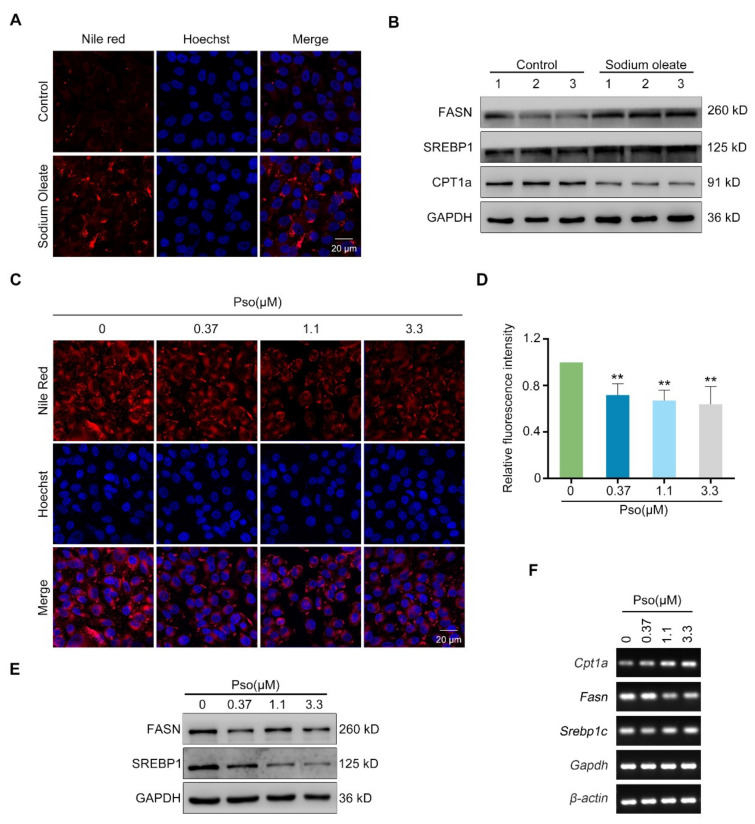
Psoralen attenuates lipid accumulation by inhibiting lipogenesis. (**A**) L02 cells were induced with sodium oleate (100 μM) for 24 h. Then lipid accumulation in cells was visualized by Nile red staining (scale bar = 20 μm). (**B**) The expressions of FASN, SREBP1, and CPT1a in control L02 cells and sodium oleate-induced L02 cells were detected by Western blotting. (**C**) L02 cells were induced with sodium oleate (100 μM) for 24 h. Then cells were treated with psoralen at 0.37, 1.1, and 3.3 μM for 24 h. The effects of psoralen on lipid accumulation in sodium oleate-induced L02 cells were determined by Nile red staining (scale bar = 20 μm). (**D**) Quantification of fluorescence intensity of Nile red staining. Quantification of FASN, SREBP1, and CPT1a protein levels. (**E**) The effects of psoralen on expressions of FASN and SREBP1 in sodium oleate-induced L02 cells were measured by Western blotting. (**F**) The effects of psoralen on the expression of FASN, SREBP1, and CPT1a in sodium oleate-induced L02 cells were measured by RT-qPCR. All values were expressed as the mean ± SD from three independent experiments. ** *p* < 0.01 vs. sodium oleate-induced group. Abbreviations: FASN, fatty acid synthase; SREBP1, sterol-regulatory element-binding protein 1; CPT1a, carnitine palmitoyltransferase 1a; Pso, psoralen.

As shown in Figure 2A, sodium oleate induced obvious lipid deposition in L02 cells as detected by Nile red staining, which may result from increased lipogenesis or reduced lipolysis. Therefore, we determined expressions of lipogenesis regulating proteins such as SREBP1 and FASN, and lipolysis rate-limiting enzyme, CPT1a, by Western blotting. As shown in Figure 2B, sodium oleate enhanced the expression of FASN and suppressed the expression of CPT1a in L02 cells. Psoralen significantly reduced lipid accumulation at 0.37, 1.1, and 3.3 μM in a dose-dependent manner (Figure 2C,D). As detected by Western blotting and RT-qPCR, Psoralen significantly decreased both the protein and mRNA levels of FASN and SREBP1 in a dose-dependent manner (Figure 2E,F). Psoralen increased the expression of CPT1a as detected by RT-qPCR.

### 3.3. Psoralen Attenuates Lipid Accumulation by Enhancing Autophagy and Promoting the Autophagic Flux

**Figure 3:**
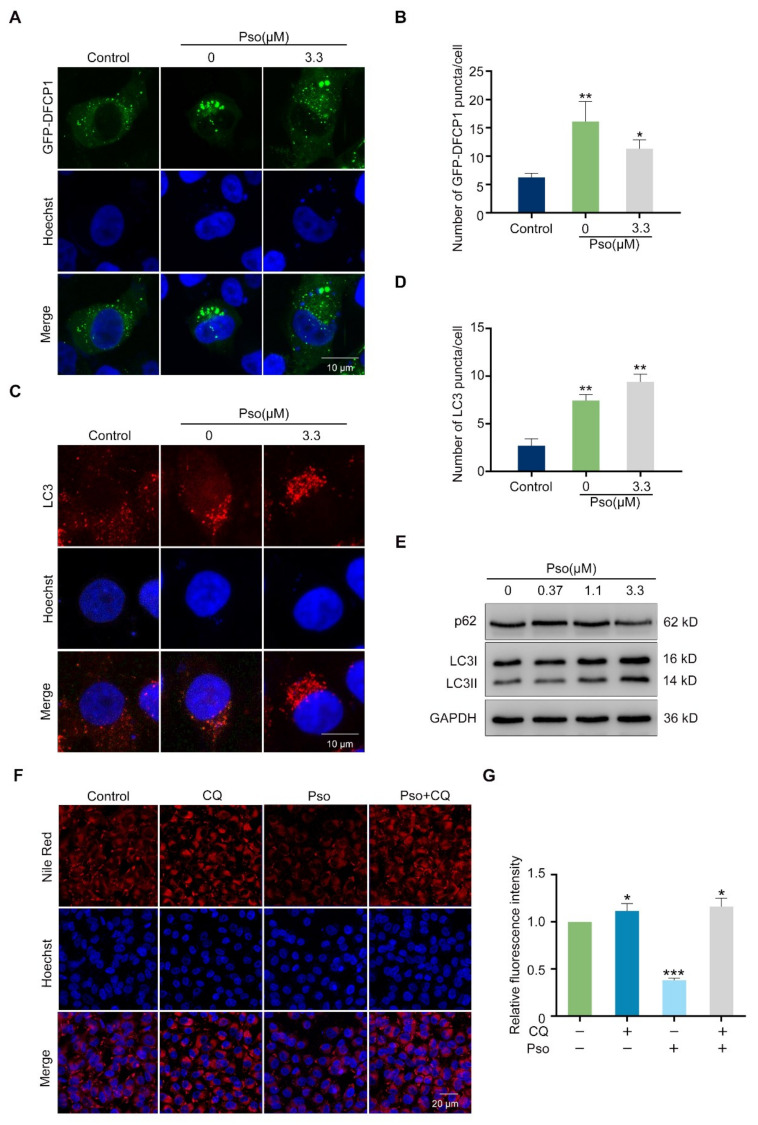
Psoralen inhibits lipid accumulation by promoting autophagy. (**A**) L02 cells were transfected with GFP-DFCP1 plasmid, induced by sodium oleate (100 μM) for 24 h, and incubated with psoralen (3.3 μM) for 24 h. Then cells were stained with Hoechst 33258 and observed by confocal microscopy (scale bar = 10 μm). (**B**) Quantification of GFP-DFCP1 puncta per cell. (**C**) The expression of LC3 was visualized by immunofluorescence and images captured by confocal microscopy (scale bar = 10 μm). (**D**) Quantification of LC3 puncta per cell. (**E**) L02 cells were induced with sodium oleate (100 μM) for 24 h to establish a NAFLD cell model. Then cells were incubated with psoralen at 0.37, 1.1, and 3.3 μM for 24 h. The expressions of p62, LC3I, and LC3II in sodium oleate-induced L02 cells were measured by Western blotting. (**F**) L02 cells were induced with sodium oleate (100 μM) for 24 h and then treated with psoralen (3.3 μM) alone or in combination with CQ (10 μM) for 24 h. Lipid accumulation was determined by Nile red staining (scale bar = 20 μm). (**G**) Quantification of fluorescence intensity of Nile red. All values were expressed as the mean ± SD from three independent experiments. * *p* < 0.05, ** *p* < 0.01, *** *p* < 0.001 vs. sodium oleate-induced group. Abbreviations: DFCP1, double FYVE-containing protein 1; CQ, chloroquine; Pso, psoralen.

**Figure 4:**
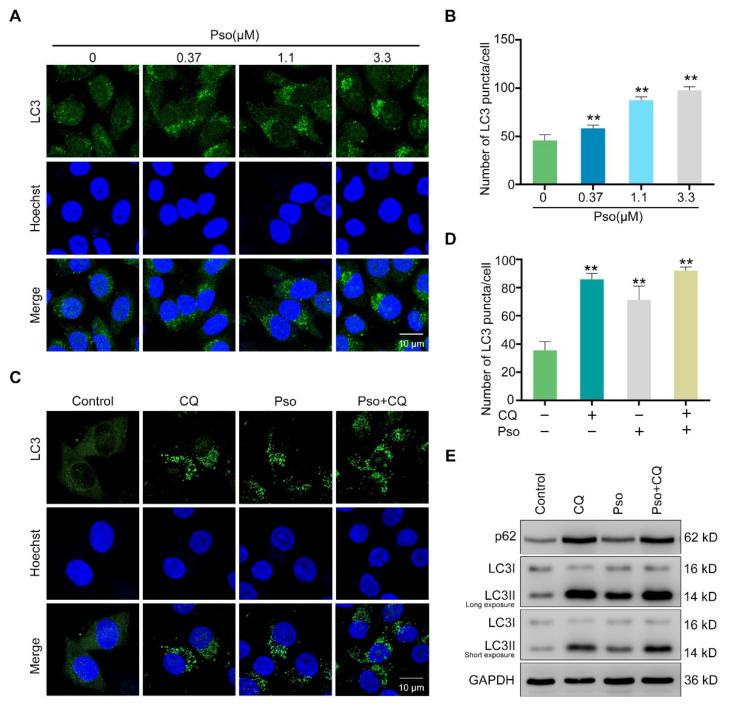
Psoralen promotes the autophagic flux. (**A**) L02 cells were induced with sodium oleate (100 μM) for 24 h and treated with psoralen at 0.37, 1.1, 3.3 μM for 24 h. The expression of endogenous LC3 was measured by immunofluorescence and observed by confocal microscopy (scale bar = 10 μm). (**B**) Quantification of LC3 puncta per cell. (**C**) L02 cells were induced with sodium oleate (100 μM) for 24 h and then treated with psoralen (3.3 μM) alone or in combination with CQ (10 μM) for 24 h. Endogenous LC3 were visualized by immunofluorescence and images captured by confocal microscopy (scale bar = 10 μm). (**D**) Quantification of LC3 puncta per cell. (**E**) The expression of p62, LC3I, and LC3II was measured by Western blotting. All values were expressed as the mean ± SD from three independent experiments. ** *p* < 0.01 vs. sodium oleate-induced group. Abbreviations: CQ, chloroquine; Pso, psoralen.

After clarifying the effects of psoralen on lipogenesis, we then focused on its impact on lipolysis. It has been reported that autophagy, as a kind of lipolysis, had important roles in human diseases resulting from lipid accumulation [13]. Therefore, we further investigated the relation between autophagy and newly generated lipid droplets by utilizing the GFP plasmids of lipid droplet marker DFCP1 [14]. As shown in Figure 3A,B, sodium oleate induced remarkable expression of DFCP1 in L02 cells, which was decreased by psoralen. Sodium oleate induced obvious endogenous LC3 puncta in L02 cells, which was further enhanced by psoralen (Figure 3C,D). To further elucidate the impact of psoralen on autophagy, we measured the ratio of LC3II/LC3I which is a reliable marker of the synthesis of the autophagosome, and the expression of the autophagy substrate, p62, which indicates the synthesis of autolysosome [15,16]. As shown in Figure 3E, psoralen significantly increased the ratio of LC3II/LC3I and decreased the expression of p62 at 0.37, 1.1, 3.3 μM in sodium oleate-induced L02 cells. Notably, CQ, the autophagy inhibitor, blocked the effects of psoralen on alleviating lipid deposition in sodium oleate-induced L02 cells (Figure 3F,G). These results demonstrated that psoralen ameliorates lipid accumulation by enhancing autophagy. Consistently, psoralen increased the number of endogenous LC3 puncta in sodium oleate-induced L02 cells in a dose-dependent manner (Figure 4A,B). Since autophagy is a continuous multistep process, we further determined the effects of psoralen on the autophagic flux and employed CQ as an autophagic flux inhibitor [17]. Both CQ and psoralen remarkably increased endogenous LC3 puncta in L02 cells. Psoralen increased endogenous LC3 puncta, which was further enhanced by CQ (Figure 4C,D). Consistently, Western blotting results showed that CQ further enhanced the ratio of LC3II/LC3I and the expression of p62 (Figure 4E).

### 3.4. Psoralen Enhances Lipolysis by Promoting the Initiation and Autophagosome-Lysosome Fusion of Autophagy

**Figure 5:**
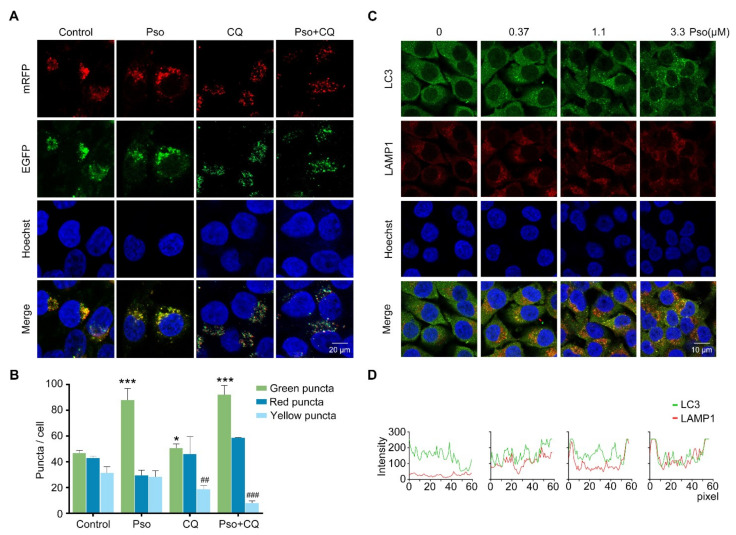
Psoralen promotes the autophagic flux by facilitating the fusion of the autophagosome and lysosome. (**A**) L02 cells were induced with sodium oleate (100 μM) for 24 h, transfected with the mRFP-EGFP-LC3 plasmid, and then treated with psoralen (3.3 μM) or CQ (10 μM) for 24 h. Nuclei were stained with Hoechst 33258 (scale bar = 20 μm). (**B**) Quantification of puncta per cell. (**C**) L02 cells were induced with sodium oleate (100 μM) for 24 h and treated with psoralen at 0.37, 1.1, 3.3 μM for 24 h, and stained with Hoechst 33258, anti-LC3, or anti-LAMP1 antibodies (scale bar = 10 μm). (**D**) Colocalization efficiency of LC3 and LAMP1 was quantified by line scan analysis (60 pixels with two ends on the membrane) by observing the overlap of fluorescence intensity peaks across the contours of multiple L02 cells (*n* ≥ 3 cells). All values were expressed as the mean ± SD from three independent experiments. * *p* < 0.05, *** *p* < 0.001 vs. sodium oleate-induced group. ## *p* < 0.01, ### *p* < 0.001, vs. psoralen treated group. Abbreviations: CQ, chloroquine; LAMP1, lysosomal associated membrane protein 1; Pso, psoralen.

**Figure 6:**
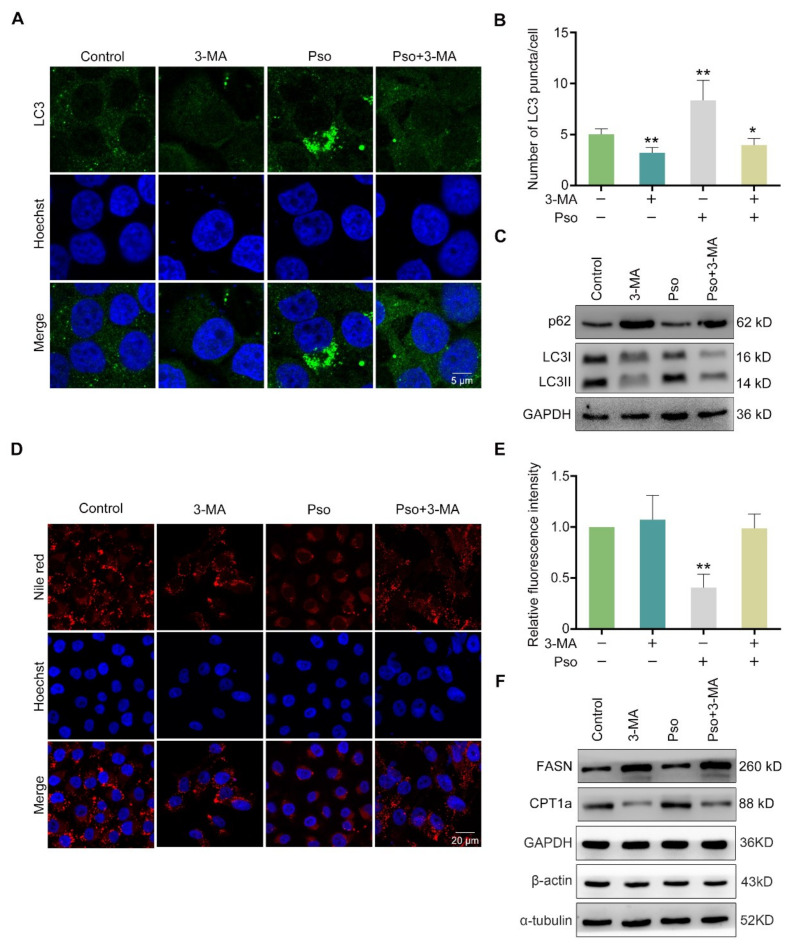
Psoralen promotes the initiation of autophagy. Sodium oleate-induced L02 cells were treated with psoralen (3.3 μM) alone or in combination with 3-MA (10 mM) for 24 h. (**A**) L02 cells were stained with the anti-LC3 antibody and Hoechst 33258 as detected by confocal microscopy (scale bar = 5 μm). (**B**) Quantification of LC3 puncta per cell. (**C**) The expression of p62, LC3I, and LC3II was measured by Western blotting. (**D**) L02 cells were stained with Nile red and Hoechst 33258 as detected by confocal microscopy (scale bar = 20 μm). (**E**) Quantification of Nile red fluorescence intensity per L02 cell. (**F**) The expression of FASN and CPT1a was measured by Western blotting. All values were expressed as the mean ± SD from three independent experiments. * *p* < 0.05, ** *p* < 0.01 vs. sodium oleate-induced control group. Abbreviations: 3-MA, 3-methyladenine; CPT1a, carnitine palmitoyltransferase 1a; Pso, psoralen; FASN, fatty acid synthase.

To further elucidate the mechanism of psoralen on the autophagic flux, we transfected L02 cells with the mRFP-EGFP-LC3 plasmid to track exogenous autophagosomes. Psoralen increased the ratio of red fluorescent mRFP-LC3 puncta to total LC3 puncta, which represents the ratio of LC3 in lysosomes to total LC3 in sodium oleosome-induced L02 cells (Figure 5A,B). Furthermore, we examined the colocalization of LC3 and LAMP1 in sodium oleate-induced L02 cells by immunofluorescence. As shown in Figure 5C, psoralen increased endogenous LC3 puncta at 0.37, 1.1, and 3.3 μM in a dose-dependent manner. As shown in Figure 5D, psoralen dose-dependently promoted the colocalization of LC3 and LAMP1 at 0.37, 1.1, and 3.3 μM. These results demonstrated that psoralen promoted the fusion of the autophagosome and lysosome. Thereafter, we wondered whether psoralen promoted autophagosome synthesis. The PI3K/AKT pathway was reported to promote autophagosome synthesis [18]. So 3-methyladenine (3-MA), the class III PI3K inhibitor, was employed. As shown in Figure 6A,B, 3-MA blocked the effects of psoralen on LC3 puncta. As detected by Western blotting, psoralen significantly decreased the expression of p62 and increased the ratio of LC3II/LC3I, which was reversed by 3-MA (Figure 6C). As detected by Nile red staining, 3-MA also blocked the effects of psoralen on lipid deposition (Figure 6D,E). Consistently, psoralen suppressed the expression of FASN and promoted the expression of CPT1a, which was reversed by 3-MA (Figure 6F). 

### 3.5. Psoralen Improves Insulin Resistance and Attenuates Lipid Accumulation by Activating AMPK

**Figure 7:**
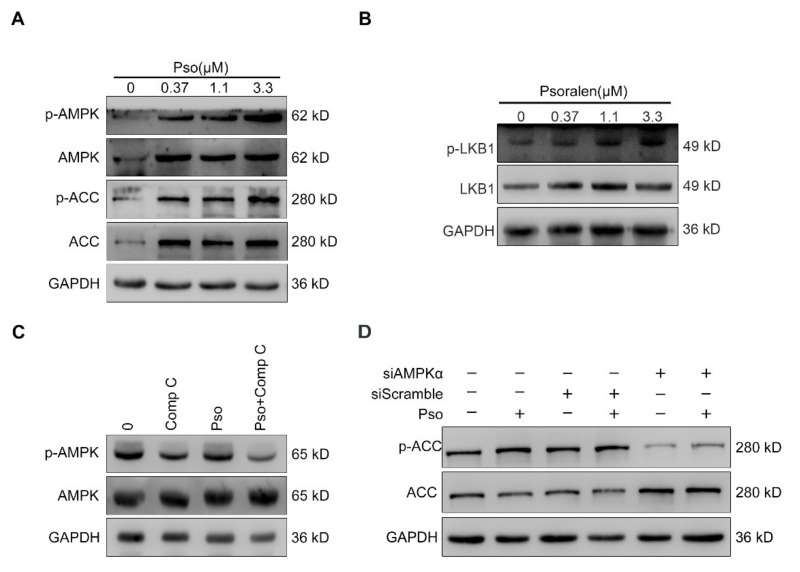
Psoralen activates the AMPK/ACC pathway by activating AMPK. (**A**) L02 cells were induced with sodium oleate (100 μM) for 24 h and treated with psoralen at 0.37, 1.1, and 3.3 μM for 24 h. The expression of AMPK, p-AMPK, ACC, and p-ACC was detected by Western blotting. (**B**) The expression of LKB1 and p-LKB1 was detected by Western blotting. (**C**) Sodium oleate-induced L02 cells were treated with psoralen (3.3 μM) alone or in combination with compound C (10 μM) for 24 h. The expression of AMPK and p-AMPK was determined by Western blotting. (**D**) L02 cells were transfected with siAMPKα or scramble, induced with sodium oleate (100 μM) for 24 h, and treated with psoralen (3.3 μM) for 24 h. The expression of ACC and p-ACC was measured by Western blotting. Abbreviations: AMPK, adenosine 5′-monophosphate (AMP)-activated protein kinase; ACC, acetyl-CoA carboxylase; Comp C, compound C; Pso, psoralen; FASN, fatty acid synthase.

**Figure 8:**
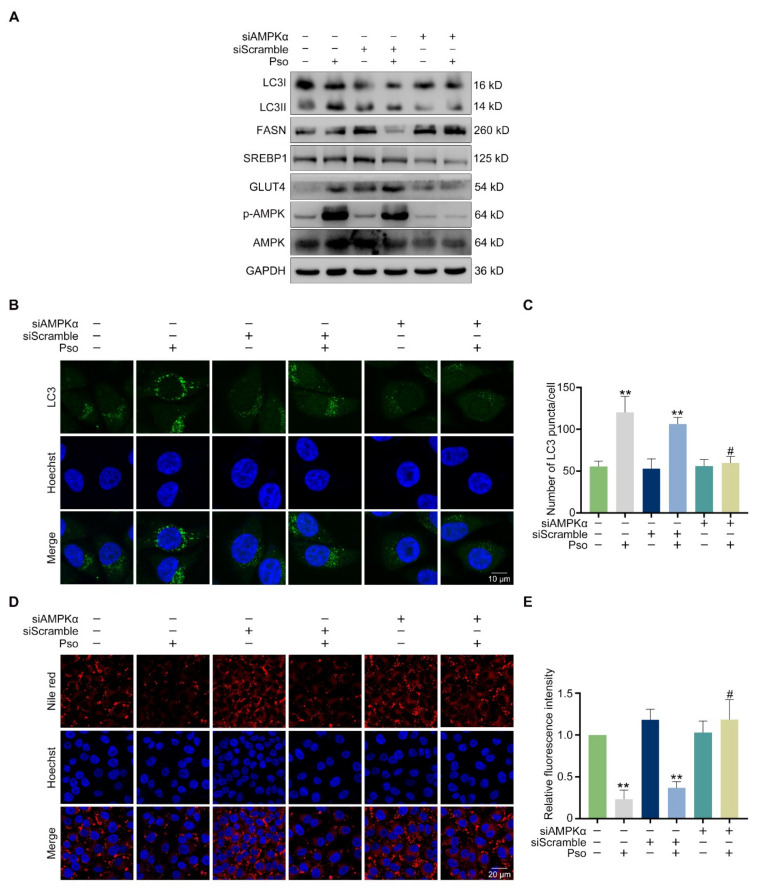
Psoralen attenuates lipid accumulation and lipogenesis via activating AMPK. L02 cells were transfected with siAMPKα or scramble, induced with sodium oleate (100 μM) for 24 h, and treated with psoralen (3.3 μM) for 24 h. (**A**) The expression of GLUT4, AMPK, p-AMPK, LC3I, LC3II, FASN, and SREBP1 was measured by Western blotting. (**B**) L02 cells were stained with anti-LC3 antibodies and Hoechst 33258 as visualized by confocal microscopy (scale bar = 10 μm). (**C**) Quantification of LC3 puncta per cell. (**D**) Lipid accumulation was determined by Nile red staining and visualized by confocal microscopy (scale bar = 20 μm). (**E**) Quantification of Nile red fluorescence intensity per cell. All values were expressed as the mean ± SD from three independent experiments. # *p* < 0.05 vs. psoralen treated group. ** *p* < 0.01 vs. sodium oleate-induced group. Abbreviations: AMPK, adenosine 5′-monophosphate (AMP)-activated protein kinase; FASN, fatty acid synthase; Pso, psoralen; FASN, fatty acid synthase; SREBP1, sterol-regulatory element-binding protein 1.

The LKB1/AMPK/ACC pathway plays a major role in promoting GLUT4 translocation, autophagy, and reducing lipogenesis [19,20,21]. LKB1 is generally regarded as the main protein activating AMPK [22]. Therefore, we wondered whether psoralen plays a role through the AMPK/ACC pathway. As shown in Figure 7A, psoralen promoted the phosphorylation of AMPK and ACC at 0.37, 1.1, and 3.3 μM in a dose-dependent manner. As shown in Figure 7B, psoralen promoted the phosphorylation of LKB1 in a dose-dependent manner. AMPKα is the catalytic subunit that is closely associated with lipogenesis [23]. Activation of AMPK by AMPKα subunit has been reported to improve energy expenditure [24]. So, we employed Compound C (Comp C) which is the AMPK inhibitor. As shown in Figure 7C, psoralen induced the phosphorylation of AMPK, which was blocked by Comp C. To further demonstrate the crucial role of the AMPKα subunit in psoralen’s effect, we knocked down AMPKα by siRNA. As detected by Western blotting, AMPKα siRNA significantly reduced the expression of AMPK (Figure 8A) and ACC (Figure 7D). Consistently, AMPKα siRNA blocked the effects of psoralen on the expression of GLUT4 (Figure 8A). Psoralen decreased the expression of FASN and SREBP1 and enhanced the ratio of LC3II/LC3I of L02 cells, which was also blocked by siAMPKα (Figure 8A). Knocking down AMPKα also significantly decreased endogenous LC3 puncta (Figure 8 B,C). As shown in Figure 8D,E, psoralen significantly alleviated lipid deposition in L02 cells, while knocking down AMPKα impaired the effects of psoralen. 

## 4. Discussion

Psoralen plus ultraviolet therapy is widely used for hyperproliferative skin disorders such as vitiligo, psoriasis, and eczema because of the high ultraviolet absorbance of psoralen [25]. Although psoralen had been reported to induce skin cancer in treating PUVA at 100 μM because of its UV-mediated activation property, we confirmed the effects of a far lower dose of psoralen (3.3 μM) on NAFLD by employing the sodium oleate-induced L02 cells [26]. Insulin resistance and hepatic lipid accumulation are the two main pathological characteristics of NAFLD. We observed that psoralen significantly ameliorated insulin resistance by upregulating the expression and membrane translocation of GLUT4 in a dose-dependent manner. Moreover, psoralen does inhibit lipogenesis by decreasing the expression of *Srebp1* and *Fasn*. Psoralen also promotes lipolysis through enhancing autophagy, whose effects were blocked by CQ or 3-MA. Our study demonstrated that psoralen does attenuate NAFLD, suggesting psoralen to be a potential candidate for NAFLD or insulin resistance-induced metabolic disorders. Considering the skin cancer-inducing effects of psoralen, NAFLD patients with hyperproliferative skin disorders or photosensitive seizures should not be given psoralen in large doses [27]. Psoralen should also be avoided to be used in combination with other medicines with phototoxicity such as doxycycline.

Various studies have indicated the tight link between NAFLD and mitochondria dysfunction, ER stress, and oxidative stress [28]. These cellular stress can be attenuated by general autophagy. Moreover, lipophagy is also a critical pathway for lipid droplet degradation. Promoting lipophagy has been demonstrated to be beneficial for alleviating lipid accumulation [13]. These reports suggested an important role of autophagy in ameliorating NAFLD. Notably, damaged autophagy has been revealed to promote NAFLD progression [29]. Promoting autophagy shall be an efficient way to ameliorate NAFLD progression. It has been reported that isopsoralen enhanced the autophagic flux to ameliorate apoptosis of rat chondrocytes [30]. However, whether psoralen improves damaged autophagy in NAFLD remains unclear. Our study demonstrated that psoralen enhanced autophagy, the initiation of autophagy, and the autophagosome-lysosome fusion in the autophagic flux in NAFLD. We employed DFCP1, which is the marker of newly generated lipid droplets. We observed the negative correlation between DFCP1-marked lipid droplet and LC3-marked autophagosome in psoralen-treated L02 cells, which indicated that psoralen enhances lipolysis by promoting autophagy. Therefore, we employed the lysosome marker, LAMP1, and the autophagosome marker, LC3, to detect the effects of psoralen on the autophagosome-lysosome fusion. Psoralen promotes the colocalization of LAMP1 and LC3, which demonstrated that psoralen promoted autophagosome-lysosome fusion. However, the increasing autophagosome-lysosome fusion may be the result of the psoralen-induced excessive endogenous autophagosome. To determine whether psoralen directly promoted the autophagosome-lysosome fusion process, we utilized mRFP-EGFP-LC3 plasmid to track exogenous autophagosomes. Psoralen increased the ratio of red mRFP-LC3 puncta/total LC3 compared to the sodium oleate-induced group. These results verified that psoralen promotes autophagosome-lysosome fusion. We also observed that psoralen enhanced total LC3 puncta compared to sodium oleate-induced L02 cells. Therefore, we concluded that psoralen promotes autophagosome synthesis. Following our hypothesis, the class III PI3K inhibitor, 3-MA, evidently blocked psoralen’s effect on promoting LC3 puncta. Notably, although psoralen decreased expression of SREBP1 and FASN, both CQ and 3-MA did suppress the effects of psoralen on alleviating lipid deposition. Our results demonstrated that psoralen promotes autophagy to alleviate lipid accumulation in NAFLD. To our knowledge, our study is the first to reveal the exact effects of psoralen on impaired autophagy in NAFLD.

AMPK has been well recognized as the key energy sensor and regulator which holds a central position in energy status [31]. The AMPK pathway plays important role in lipid and glucose metabolism, and autophagy [21]. The activity of AMPK is reduced by obesity, inflammation, and diabetes [32]. Enhancing the activity of AMPK has been considered as a feasible strategy for ameliorating NAFLD [33]. Our study showed that psoralen attenuates lipid deposition in sodium oleate-induced L02 cells by activating the LKB1/AMPK/ACC pathway. Notably, knocking out the AMPKα subunit by siRNA blocked the effects of psoralen on the expression of *Srebp1*, *Fasn*, and autophagy. Most importantly, knocking out the AMPKα subunit did reverse the inhibition of psoralen on lipid accumulation. In addition to NAFLD, chronic energy imbalance leads to many metabolic diseases such as obesity and type 2 diabetes mellitus [34,35]. Activating AMPK enhances energy expenditure, which helps to restore energy balance. So, our study suggested that psoralen may be a potential candidate for these metabolic diseases by activating AMPK.

The lipid accumulation in hepatocytes has been reported partly due to defects in lipoprotein synthesis and secretion [36]. Increased lipid peroxidation may disrupt the membrane structure in cells such as endoplasmic membrane and mitochondria and lead to lipid metabolism disorders [37]. As an antioxidant, psoralen may play lipid-lowering effects by decreasing lipid peroxidation. Psoralen was reported to reduce the expression of cyclooxygenase-2 (COX-2) and inducible nitric oxide synthase (iNOS) in lipopolysaccharide (LPS)-induced mouse macrophages (RAW264.7) in vitro [38]. Therefore, we believe that psoralen may attenuate NAFLD by ameliorating lipid deposition, decreasing lipid peroxidation, and promoting the expression of COX-2 and iNOS. 

Although no specific drugs have been approved by the FDA for NAFLD, some promising medicines such as Sirtuin 4 (SIRT4) targeting drugs are already in the pipeline [39]. SIRT4 has been identified as a key molecule in NAFLD. SIRT4 coordinates the balance between lipid synthesis and catabolism [40]. More importantly, SIRT4 regulates fatty acid oxidation and mitochondrial gene expression in both hepatocytes and muscle cells. These characteristics make SIRT4 a pharmacological target for NAFLD since NAFLD is a complicated metabolic disease involving the liver, muscle, and adipose [41]. Enhanced circulating levels of SIRT4 have been identified as a potential marker for oxidative stress in NAFLD patients [42]. Natural products such as flavonoids have been extensively demonstrated as modulators of sirtuins including SIRT4 [43]. As a kind of flavonoid, psoralen may also be a potential modulator of SIRT4, which needs to be verified in further studies.

Our study has some limitations. The effects of psoralen for NAFLD need to be further confirmed by animal experiments and clinical trials. Furthermore, the binding of psoralen to the AMPKγ1 subunit was determined by molecular docking and siRNA, which will be strengthened by a co-immunoprecipitation assay.

In conclusion, our study demonstrated that psoralen, as an antioxidant, ameliorates NAFLD by alleviating insulin resistance and restoring lipid metabolism homeostasis. Psoralen exerts effects by promoting autophagosome synthesis and the autophagic flux via activating AMPK. Our study suggested that psoralen is a promising candidate for metabolic diseases by activating AMPK to enhance energy expenditure and alleviate insulin resistance.

## Data Availability

All data are available upon request from the corresponding author.

## Supplementary Materials

The following supporting information can be downloaded at: https://www.mdpi.com/article/10.3390/cells11071067/s1; Table S1: RT-qPCR primers of *Cpt1a*, *Fasn*, *Srebp1c*, *Gapdh*, *β-actin*.
