# Supplementary material for: Psoralen Suppresses Lipid Deposition by Alleviating Insulin Resistance and Promoting Autophagy in Oleate-Induced L02 Cells
Source: Cells. 2022 Mar 22;11(7):1067. doi: 10.3390/cells11071067 (PMC8997557; doi:10.3390/cells11071067)
Supplement: Supplementary file 1 [file cells-11-01067-s001.zip › cells-1598853-supplementary.pdf]

Supplementary Table S1

Table S1. RT-qPCR primers of *Cpt1a, Fasn, Srebp1c, Gapdh, β-actin*.

Gene name	Forward primer(5'to3')	Reverse primer(5'to3')
Cpt1a	CTACACGGCCGATGTTACGA	GCCTTTGCAGTGCCCATC
Fasn	GTCCACCAGCAACATCAGC	GTTCTCCAGCAAGCCATCTC
Srebp1c	TGAGGACAGCAAGGCAAAG	CAGGACAGGCAGAGGAAGAC
Gapdh	GGAGCGAGATCCCTCCAAAAT	GGCTGTTGTCATACTTCTCATGG
β-actin	CATGTACGTTGCTATCCAGGC	CTCCTTAATGTCACGCACGAT
α-tubulin	CCACAGTCATTGATGAAGTTCG	GCTGTGGAAAACCAAGAAGC
